# Pregnancy Loss History Is Associated with Systemic Involvement and Disease Activity in Women with Behçet’s Disease: A Retrospective Cohort Study

**DOI:** 10.3390/diagnostics16081133

**Published:** 2026-04-10

**Authors:** Rui Bu, Yan Ma, Xinyu Li, Qiu Li, Liangjing Lu

**Affiliations:** Department of Rheumatology, Renji Hospital, Shanghai Jiao Tong University School of Medicine, Shanghai 200001, China; buruiii@163.com (R.B.); my151220@163.com (Y.M.); lixinyu1013@126.com (X.L.); liqiu1020@sjtu.edu.cn (Q.L.)

**Keywords:** Behçet’s disease, pregnancy loss, miscarriage, systemic involvement, disease activity, reproductive outcomes

## Abstract

**Background/Objectives:** Behçet’s disease (BD) is a systemic vasculitis frequently affecting women of childbearing age. However, the relationship between systemic manifestations and pregnancy loss remains unclear. This study evaluated the association between pregnancy loss history and systemic clinical characteristics in women with BD. **Methods:** This retrospective cohort study included 114 women with BD followed in a rheumatology outpatient clinic between January 2021 and December 2025. In total, 196 pregnancies were recorded. Women without a pregnancy history were excluded. Pregnancy loss was defined as any spontaneous loss, including biochemical pregnancy, miscarriage, or fetal death, excluding elective terminations. Disease activity was assessed using the Krause score, and univariable logistic regression was performed. **Results:** Among 97 women with a pregnancy history, 25 (25.8%) had at least one pregnancy loss. Compared with women without pregnancy loss, those with pregnancy loss had longer disease duration and higher Krause scores. Gastrointestinal involvement (OR 6.31, 95% CI 1.87–23.28, *p* = 0.0035) and ocular involvement (OR 3.93, 95% CI 1.44–10.89, *p* = 0.0076) were significantly associated with pregnancy loss history. Higher Krause scores were also associated with greater odds of pregnancy loss history. **Conclusions:** In women with BD, pregnancy loss history was associated with systemic organ involvement and higher disease activity, particularly gastrointestinal and ocular involvement. These findings should be interpreted cautiously in light of the retrospective design and univariable analyses, and they suggest that pregnancy loss history may be associated with greater systemic disease burden.

## 1. Introduction

Behçet’s disease (BD) is a chronic, recurrent multisystem vasculitis characterized by recurrent oral and genital ulcers, skin lesions, and varying degrees of ocular, gastrointestinal, neurological, and vascular involvement [1,2]. Epidemiologically, BD shows marked geographic variation and is most prevalent in countries along the ancient “Silk Road” [3]. A review of population-based epidemiological studies reported a pooled global prevalence of approximately 10.3 per 100,000 [4]. Because the disease is usually diagnosed during the childbearing years [3], pregnancy-related outcomes have become an important clinical concern.

Previous studies evaluating pregnancy outcomes in women with BD have reported inconsistent results [5]. Some studies suggest that the pregnancy outcomes in women with BD are similar to those in healthy controls [6,7], whereas others report an increased risk of miscarriage, fetal growth restriction, and other obstetric complications [8,9]. However, these studies have differed not only in their findings, but also in their analytic frameworks and outcome definitions. Many reports have evaluated pregnancy-level outcomes or composite adverse pregnancy outcomes, whereas fewer studies have examined pregnancy loss as a distinct patient-level clinical history. These discrepancies may be influenced by differences in disease phenotype and overall severity, particularly the presence and extent of systemic organ involvement. Therefore, it remains unclear whether a history of pregnancy loss identifies a subgroup of women with BD with a more severe systemic phenotype.

Pregnancy represents a unique immune and vascular state. Chronic systemic inflammation, endothelial dysfunction, and prothrombotic tendencies are potential mechanisms linking systemic inflammatory diseases to adverse pregnancy outcomes [10,11]. BD is a systemic vasculitis that involves blood vessels of different sizes, which may cause endothelial damage and coagulation abnormalities [12]. These processes may theoretically impair placental vascular function and increase the risk of pregnancy loss, although direct clinical evidence supporting these mechanisms in BD remains limited. In this context, pregnancy loss history may reflect not only a reproductive event, but also a marker of broader systemic inflammatory and vascular burden in BD.

Importantly, BD is not just a mucocutaneous disease, but a systemic inflammatory vasculitis. Major-organ involvement may indicate a greater overall inflammatory burden and a more severe systemic phenotype in BD [3]. However, it remains unclear whether pregnancy loss history at the patient level is associated with greater organ involvement and a higher overall disease activity burden in women with BD.

Therefore, this study aimed to evaluate whether pregnancy loss history, considered as a patient-level clinical history, is associated with greater organ involvement and higher overall disease activity burden in women with BD. By shifting the focus from pregnancy-level outcomes to patient-level reproductive history, this study seeks to clarify the clinical relevance of pregnancy loss as a potential marker of systemic disease severity and to provide a more phenotype-oriented perspective on reproductive outcomes in BD.

## 2. Methods

### 2.1. Study Design and Participants

This study was a retrospective secondary analysis based on the Pregnant women with Connective tissue disease Cohort for Outcomes Study (PCCOS). PCCOS is an ongoing prospective cohort study that has enrolled 1406 pregnant women with rheumatic diseases. Under a unified research framework, it prospectively collects pregnancy-related follow-up data, outcome records, and outpatient pregnancy information. The study was approved by the Institutional Review Committee of Renji Hospital affiliated with Shanghai Jiaotong University (Ethics No. [2017]201), and complied with the institutional data protection policy. Data were extracted from the hospital electronic medical record system, including laboratory and imaging reports, as well as outpatient follow-up data. All data were de-identified before statistical analysis.

Within the PCCOS framework, women of childbearing age (18–50 years) with BD who were evaluated and followed at the outpatient clinic of the Department of Rheumatology and Immunology, Renji Hospital, between January 2021 and December 2025 were screened for eligibility. The diagnosis of BD was based on the 1990 International Study Group criteria. All eligible women who met these criteria and had sufficient information on clinical manifestations and pregnancy history in their medical records during the study period were included. Ultimately, 114 women were included, contributing a total of 196 pregnancies. The study size was determined by all eligible women with Behçet’s disease who met the inclusion criteria within the PCCOS framework during the study period, rather than by a pre-specified sample size calculation. Because this was a retrospective analysis of an existing clinical cohort, no formal sample size estimation was performed in advance. Women without a pregnancy history (G0P0) were excluded from the pregnancy outcome analysis. When pregnancy outcome records were incomplete, the information was verified by structured telephone follow-up.

### 2.2. Definition of Pregnancy Loss and Clinical Variables

The primary outcome was patient-level pregnancy loss history, which was defined as at least one spontaneous pregnancy loss. Pregnancy loss included biochemical pregnancy, miscarriage, or fetal death. Elective termination of pregnancy was not considered a pregnancy loss event and was excluded from the outcome classification. For women with multiple pregnancies, pregnancy loss history was defined according to whether they had experienced at least one spontaneous pregnancy loss, and individual pregnancies were not treated as independent observations. Pregnancy loss was ascertained primarily from medical records and was additionally verified by outpatient documentation or structured telephone follow-up when necessary, in order to reduce outcome misclassification. Clinical variables used in the primary analysis were obtained from routine outpatient records and medical history documentation within the same clinical framework.

Clinical manifestations were classified according to organ involvement, including mucocutaneous manifestations, joint involvement, gastrointestinal involvement, ocular involvement, central nervous system involvement, cardiovascular involvement, and thrombosis history. Mucocutaneous involvement mainly included oral ulcers, genital ulcers, and skin lesions, which were recorded and analyzed as independent variables. Organ involvement was determined based on specialist documentation in the medical records and confirmed by relevant examination results. For example, ocular involvement was confirmed by ophthalmologic evaluation; gastrointestinal and central nervous system involvement were confirmed by endoscopy, imaging studies, neurological assessment, or other appropriate objective findings. Thrombotic and cardiovascular events were recorded based on clinically documented diagnoses supported by objective evidence.

Disease activity was assessed using the Krause score [13]. The most recent outpatient Krause score recorded during the study period (2021–2025) was used to reflect the overall disease activity burden and systemic inflammatory status during follow-up. Because some pregnancy events occurred before the study period, the Krause score was not interpreted as a measure of disease activity at the time of pregnancy. Gestational hypertension [14], gestational diabetes mellitus [15], and preterm birth [16] were defined according to the current obstetric guidelines.

### 2.3. Outcomes

The main objective was to assess the association of patient-level pregnancy loss history with systemic manifestations and disease activity in BD. Secondary analyses included other adverse pregnancy outcomes and exploratory comparisons of laboratory, immunological, coagulation, and lymphocyte subset parameters.

### 2.4. Statistical Analysis

Continuous variables with a normal distribution were expressed as mean ± standard deviation (SD), whereas non-normally distributed variables were expressed as median and interquartile range (IQR). Categorical variables were summarized as counts and percentages. For between-group comparisons, the Wilcoxon rank-sum test was used for continuous variables and Fisher’s exact test for categorical variables. Given the limited number of outcome events, the analysis was restricted to univariable logistic regression to evaluate the association between pregnancy loss history and clinical variables, including systemic organ involvement and Krause score. Odds ratios (ORs) and 95% confidence intervals (CIs) were calculated. All statistical analyses were performed using R software (version 4.5.1), and a two-sided *p*-value < 0.05 was considered statistically significant. The primary analyses were based on core clinical variables, including systemic manifestations, disease activity, pregnancy history, and obstetric outcomes. Missing data mainly affected laboratory, immunological, coagulation, HLA, and lymphocyte-related variables, which were included only in exploratory analyses. Therefore, no imputation was performed.

## 3. Results

### 3.1. Study Population and Overall Pregnancy Outcomes

A total of 114 women of reproductive age with Behçet’s disease (BD) were included in this study, contributing 196 documented pregnancies. The mean age of the overall cohort was 34.4 ± 5.5 years, with a mean age at disease onset of 25.9 ± 7.0 years and a median disease duration of 7.0 years (IQR 10.0) (Table 1). Mucocutaneous manifestations were the most common clinical features, including oral ulcers, genital ulcers, and skin lesions. Among systemic manifestations, ocular, joint, and gastrointestinal involvement were the most frequently observed (Table 1).

**Figure 1:**
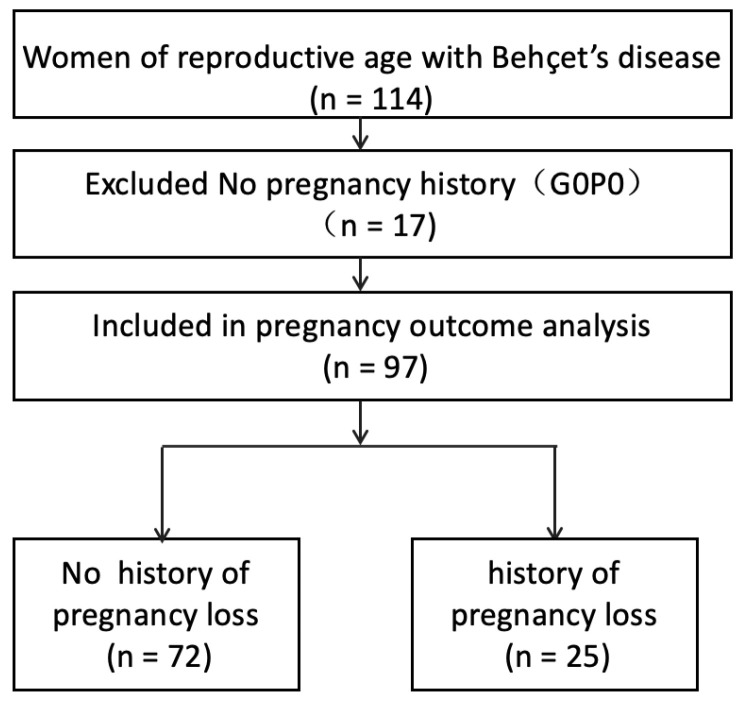
Flowchart of patient inclusion and pregnancy outcome analysis. G0P0 indicates nulligravida (no pregnancy history).

Regarding reproductive history, 97 women (85.1%) had experienced at least one pregnancy and were therefore included in the pregnancy outcome analysis (Figure 1). Among these women, 25 (25.8%) had at least one spontaneous pregnancy loss. Overall, 90 women had at least one live birth, whereas 7 had no history of live birth because all pregnancies resulted in pregnancy loss. Because pregnancy complications such as gestational hypertension, gestational diabetes mellitus, and mode of delivery can only be evaluated among pregnancies progressing to delivery, analyses of these outcomes were restricted to women with at least one live birth (*n* = 90).

### 3.2. Clinical Characteristics Stratified by Pregnancy Loss History

Among the 97 women with a pregnancy history, 72 had no history of pregnancy loss, and 25 had experienced at least one pregnancy loss (Table 2). There were no significant differences between the two groups with respect to age, age at disease onset, or age at first pregnancy.

**Figure 2:**
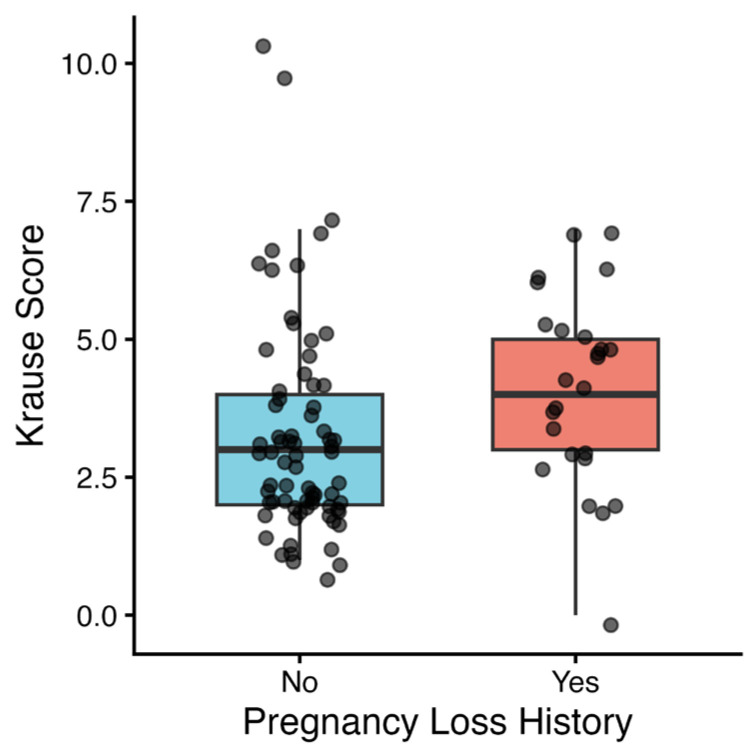
Krause disease activity scores stratified by pregnancy loss history in women with Behçet’s disease.

Compared with women without pregnancy loss, women with a history of pregnancy loss had a significantly longer disease duration (*p* = 0.031) and higher Krause scores (*p* = 0.011) (Figure 2). Ocular involvement (44% vs. 17%, *p* = 0.012) and gastrointestinal involvement (32% vs. 6.9%, *p* = 0.004) were significantly more frequent in women with pregnancy loss. No statistically significant differences were observed for other clinical manifestations, including mucocutaneous manifestations, joint involvement, central nervous system involvement, history of thrombosis, or cardiovascular involvement (Table 2).

As a secondary analysis, clinical characteristics were also compared according to the presence of adverse pregnancy outcomes (APO) (Supplementary Table S2). These findings were considered supportive and were not used as the basis for the primary conclusions.

### 3.3. Association Between Pregnancy Loss History and Systemic Involvement

**Figure 3:**
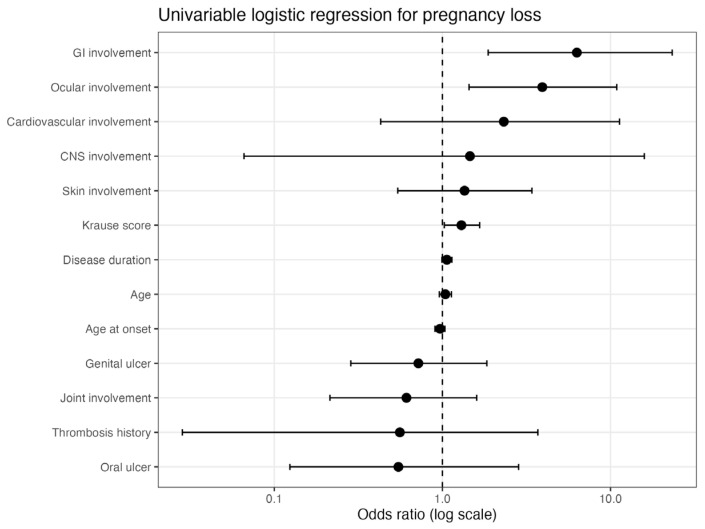
Forest plot of univariable logistic regression results for factors associated with pregnancy loss history.

To further evaluate the association between pregnancy loss history and systemic disease manifestations, univariable logistic regression analyses were performed (Table 3; Figure 3). Gastrointestinal involvement showed a strong association with pregnancy loss history (OR 6.31, 95% CI 1.87–23.28, *p* = 0.004). Similarly, ocular involvement was significantly associated with pregnancy loss history (OR 3.93, 95% CI 1.44–10.89, *p* = 0.008). A higher Krause score was also associated with pregnancy loss history (OR 1.30 per point increase, 95% CI 1.03–1.67, *p* = 0.033). In contrast, demographic variables, including age and age at disease onset, were not significantly associated with pregnancy loss history. Likewise, mucocutaneous manifestations, joint involvement, central nervous system involvement, thrombosis history, and cardiovascular involvement did not show statistically significant associations. Although the odds ratios for central nervous system and cardiovascular involvement were greater than 1, their confidence intervals were wide and crossed unity, suggesting considerable statistical uncertainty due to the small number of cases.

Overall, the univariable analyses indicate that systemic organ involvement, particularly gastrointestinal and ocular involvement, as well as higher disease activity burden, were associated with pregnancy loss history in women with BD.

### 3.4. Exploratory Analysis of Laboratory, Immunological, Coagulation, and Lymphocyte Subset Parameters

Laboratory, immunological, coagulation, HLA, and lymphocyte-related variables were not available for all participants; therefore, these analyses were exploratory and based on available data only (Supplementary Table S1). Exploratory comparisons of laboratory, immunological, coagulation, and lymphocyte subset parameters between women with and without pregnancy loss history are presented in Supplementary Table S3. No consistent differences were observed between the two groups. Because these parameters were not available for all patients and were measured at non-standardized time points, the results should be interpreted as exploratory.

## 4. Discussion

Behçet’s disease (BD) is a chronic multisystem vasculitis that commonly affects women of childbearing age. Accordingly, pregnancy-related outcomes have become an important clinical concern. In this study cohort, the mean age at disease onset was 25.9 ± 7.0 years, consistent with previous epidemiological reports. As expected, mucocutaneous involvement, especially oral ulcers and genital ulcers, was the most common clinical feature [17,18]. Beyond describing the baseline characteristics, we found that patient-level pregnancy loss history was associated with systemic organ involvement and a higher overall disease activity burden. Overall, these results suggest that the history of pregnancy loss in women with BD may be a clinical marker of higher systemic inflammatory burden, rather than isolated obstetric events.

Previous studies have reported inconsistent pregnancy outcomes in women with Behçet’s disease, ranging from outcomes comparable to healthy controls to increased risks of miscarriage, fetal growth restriction, and other obstetric complications [19]. Such variability likely reflects not only differences in outcome definition and analytic framework, but also heterogeneity in disease phenotype across cohorts. In particular, variation in systemic involvement profiles across cohorts may reflect differences in overall phenotype severity and thereby influence reproductive risk. This issue may be especially relevant in regions along the ancient Silk Road, where Behçet’s disease is more prevalent, and phenotype distributions differ across populations. Previous studies from high-prevalence regions, including China and other Silk Road-associated settings, have likewise reported heterogeneous pregnancy outcomes, suggesting that regional variation in systemic involvement, disease severity, referral patterns, and clinical management may contribute to inconsistent findings across cohorts [20]. In addition, definitions and timing of disease activity assessment also vary across studies, with some using pregnancy-anchored activity measures and others relying on cross-sectional or follow-up assessments. In this context, our study provides complementary evidence by examining pregnancy loss history as a distinct patient-level outcome in relation to systemic organ involvement and overall disease activity burden in women with Behçet’s disease.

A biologically plausible link between systemic vasculitis and adverse reproductive outcomes has been suggested in prior literature [10], although the specific pathways discussed below were not directly assessed in the present cohort. Successful implantation and placental development depend on a series of immune and vascular adaptations in early pregnancy, and this stage is particularly sensitive to disturbances in maternal vascular and immune homeostasis. The coordinated processes of trophoblast invasion, spiral artery remodeling, and local immune tolerance are key steps in early placentation [21]. In the setting of systemic inflammation, cytokine imbalance and endothelial/microvascular injury may disrupt these processes, thereby increasing the risk of early pregnancy loss [22]. Meanwhile, pregnancy itself remodels the endocrine and immune environment, and the maternal-fetal interface and peripheral immune response show a shift toward Th2 polarization [23,24]. Previous studies have indicated that this immune shift may be associated with decreased activity or symptom relief in predominantly Th1-driven vasculitides (potentially including BD), whereas vasculitides with more prominent Th2-related pathways (such as GPA and EGPA) may follow different clinical trajectories [25]. However, even if the clinical symptoms during pregnancy tend to be relieved, BD-related endothelial dysfunction and microvascular inflammatory burden may persist and could potentially contribute to pregnancy loss through impaired placental vascular function and early placentation. Based on this biological framework, key pathophysiological characteristics of BD (neutrophil-driven inflammation, endothelial injury, and vasculitis involving vessels of different sizes) may exacerbate microvascular dysfunction and inflammatory signals [26,27]. Against this background, it is plausible that pregnancy loss history may co-occur with a higher systemic inflammatory burden at the phenotypic level, although this mechanism was not directly examined in the present cohort. Based on the research design and available data, our findings are best interpreted as a phenotypic association: pregnancy loss history tends to co-occur with systemic involvement and higher systemic inflammatory burden, rather than providing evidence of event-level causality. This interpretation is also consistent with prior literature on systemic vasculitis, in which greater inflammatory burden has been linked to early pregnancy loss and other adverse outcomes [25].

The observed associations of gastrointestinal and ocular involvement with pregnancy loss history are clinically relevant and broadly consistent with prior clinical and pathophysiological literature. Gastrointestinal involvement in BD is often associated with high inflammatory activity and systemic immune activation [28]. It is also frequently accompanied by deep ulcers and extensive mucosal damage [29]. These interpretations are based on prior mechanistic and clinical literature rather than direct measurement in the present study. Based on prior literature, this inflammatory environment may affect early pregnancy through a variety of non-mutually exclusive pathways, including impaired endometrial receptivity [30] and early placental microvascular dysfunction [31]. Gastrointestinal involvement often coexists with broader systemic activity and may serve as a marker of disease severity. Against this background, the association between gastrointestinal involvement and pregnancy loss history may be understood in relation to the broader systemic inflammatory burden that gastrointestinal involvement often reflects. Similarly, ocular involvement reflects vasculitic processes affecting both arteries and veins and is often considered a sign of more severe systemic disease [32,33]. Immune-mediated endothelial injury is a central driver of vascular pathology in BD [34]. In parallel, neutrophil-mediated vasculitis has been recognized as another major pathogenic process contributing to vascular involvement in this disease [35,36]. From a reproductive perspective, prior literature suggests that these systemic vasculitic processes may impair placental perfusion, thereby increasing susceptibility to early pregnancy loss. Clinically, gastrointestinal and ocular involvement are also often regarded as alternative indicators of systemic severity and inflammatory burden [18,37], which may help contextualize their association with pregnancy loss history in the present study. In view of the limited number of events, these findings should still be interpreted with caution.

In addition to gastrointestinal and ocular involvement, another finding worth discussing is the association between the Krause score and pregnancy loss history. The Krause score was derived from the most recent available outpatient assessment during the study period (2021–2025) and was intended to reflect the overall disease activity burden, rather than pregnancy-anchored disease activity. Because some pregnancy losses occurred many years before the study period, the Krause score should not be regarded as a pregnancy-anchored exposure measure. Rather, we regarded the Krause score as a pragmatic indicator of phenotype severity to describe the overall inflammatory burden and multisystem activity of patients during follow-up. Within this framework, the association between a higher Krause score and pregnancy loss history may indicate that women with such a history tend to have a greater systemic disease burden during follow-up. In the future, if standardized activity assessments can be collected before conception and during pregnancy, it will help to determine whether pregnancy-anchored activity assessment provides additional predictive information beyond the phenotype severity of the follow-up period. There is no significant association between the thrombosis history and pregnancy loss history in this study, which also needs to be interpreted with caution. In many systemic diseases, thrombosis and hypercoagulability are important factors leading to adverse pregnancy outcomes [38,39]. However, thrombosis in BD is different from classical hypercoagulable disorders [40]. It is increasingly recognized as an inflammation-driven process mainly related to endothelial dysfunction and vasculitis [41], and may be better understood as part of an inflammatory-thrombotic state rather than an isolated coagulation abnormality [42]. Against this background, systemic inflammatory activity may be more closely related to reproductive risk than thrombosis history itself, although this inference cannot be directly established from the present data. Given the small number of thrombosis events in this single-center cohort and the resulting limited statistical precision, the absence of a significant association should not be taken to exclude a contribution of vascular factors to reproductive risk; rather, it may reflect a more dominant role of systemic inflammatory burden.

From a clinical perspective, our findings suggest that women with BD should ideally conceive during disease remission and optimize the control of systemic disease before conception [43]. A history of pregnancy loss may serve as a clinical risk indicator, prompting a more comprehensive systemic assessment, with particular attention to the gastrointestinal and ocular involvement and the overall inflammatory burden. These findings also support the importance of multidisciplinary management to provide more individualized monitoring and care for women planning pregnancy. Overall, women with a history of pregnancy loss and systemic organ involvement may represent a higher-risk subgroup and require closer follow-up.

This study has several strengths. We used a clear definition of patient-level pregnancy loss history, including biochemical pregnancy, miscarriage, and fetal death, and excluded elective terminations, thereby focusing on spontaneous pregnancy loss as a distinct reproductive endpoint. We performed detailed phenotyping of systemic organ involvement, enabling assessment of organ-specific associations beyond mucocutaneous manifestations. In addition, we used the Krause score as a standardized measure of overall disease activity burden during follow-up. This allowed us to examine its association with pregnancy loss history as an indicator of phenotype severity, rather than as a pregnancy-anchored activity measure. Finally, the consistency of the group comparisons and the results of univariable regression support the credibility of the observed associations.

Several limitations should be acknowledged. The retrospective single-center design may introduce selection bias and limit its generalizability. Pregnancy outcomes were primarily ascertained from medical records and supplemented by telephone follow-up, which may be affected by recall bias. The sample size, especially for the organ-involved subgroup, was relatively small, resulting in wide confidence intervals for some estimates. Accordingly, the regression results should be interpreted as unadjusted associations, and the absence of multivariable modeling limits the ability to account for potential confounding by age, disease duration, treatment exposure, and other factors. In addition, medication exposure before conception and during the pregnancies of interest could not be reliably assessed, because many patients were diagnosed with Behçet’s disease only after pregnancy or after pregnancy loss. Therefore, the potential influence of treatment and time-varying disease activity during pregnancy could not be evaluated in a pregnancy-anchored manner. Moreover, the Krause score reflected disease activity during follow-up rather than at the time of pregnancy, which may have introduced temporal misalignment between exposure assessment and pregnancy loss events. Collectively, these limitations warrant cautious interpretation of our findings.

Future studies should validate these findings in larger prospective, multicenter cohorts with standardized longitudinal assessment of disease activity and treatment exposure before conception and throughout pregnancy. When feasible, pregnancy-level analyses and integration of placental pathology or vascular-inflammatory biomarkers may help clarify underlying pathways and improve risk stratification for women with BD planning pregnancy.

## 5. Conclusions

This study shows that pregnancy loss history in women with BD is associated with systemic organ involvement and a higher overall disease activity burden, particularly gastrointestinal and ocular involvement. These findings suggest that pregnancy loss history may be a clinical marker of greater systemic disease burden and may support more comprehensive clinical assessment in women of childbearing age with BD. Therefore, identifying a history of pregnancy loss may provide meaningful insights for clinical practice, help assess and manage the severity of the disease in women of childbearing age, and support a more comprehensive clinical evaluation and management strategy. Further prospective studies are warranted to confirm these observations.

## Figures and Tables

**Table 1:** Baseline characteristics of women with Behçet’s disease.

	Behçet’s Disease (*n* = 114)
**Demographics**	
Age (years)—mean (±SD)	34.4 ± 5.5
Age at onset (years)—mean (±SD)	25.9 ± 7.0
Disease duration (years)—median (IQR)	7.0 (10.0)
Age at first pregnancy (years)—mean (±SD)	26.3 ± 4.6
**Clinical manifestations**—*n* (%)	
Oral ulcers	105/114 (92.1)
Genital ulcers	71/114 (62.3)
Skin involvement	62/114 (54.4)
Ocular involvement	27/114 (23.7)
Joint involvement	41/114 (36.0)
Gastrointestinal involvement	25/114 (21.9)
CNS involvement	5/114 (4.4)
Thrombosis history	7/114 (6.1)
Cardiovascular involvement	6/114 (5.3)
**Disease activity (available)**	
Krause score—median (IQR)	3.0 (3.0)
**Obstetric history**—*n* (%)	
No pregnancy history (G0P0)	17/114 (14.9)
Ever pregnant	97/114 (85.1)
Pregnancy loss history	25/97 (25.8)
1	16/97 (16.5)
≥2	9/97 (9.3)
**Pregnancy complications**—*n* (%)	
Gestational hypertension	6/90 (6.7)
Gestational diabetes mellitus	7/90 (7.8)
Cesarean section	36/90 (40.0)
Vaginal delivery	52/90 (57.8)
Both modes across pregnancies	2/90 (2.2)

Data are presented as mean ± standard deviation (SD), median (interquartile range, IQR), or number (percentage).

**Table 2:** Clinical characteristics of women with Behçet’s disease according to pregnancy loss history.

Variable	No History of Pregnancy Loss (*N* = 72)	History of Pregnancy Loss (*N* = 25)	*p*-Value
Age	33.50 (31.00–38.50)	36.00 (32.00–38.00)	0.3
Age at onset	27.00 (21.00–30.25)	27.00 (21.00–29.00)	0.4
Disease duration	6.50 (2.00–12.00)	9.00 (6.00–15.00)	0.031
Krause score, median (IQR)	3.0 (2.0–4.0)	4.0 (3.0–5.0)	0.011
Age at first pregnancy	27.00 (23.00–30.00)	27.00 (25.00–30.00)	0.6
Oral ulcers	67 (93%)	22 (88%)	0.4
Genital ulcers	46 (64%)	14 (56%)	0.5
Skin involvement	32 (44%)	13 (52%)	0.6
Ocular involvement	12 (17%)	11 (44%)	0.012
Gastrointestinal involvement	5 (6.9%)	8 (32%)	0.004
Joint involvement	28 (39%)	7 (28%)	0.5
CNS involvement	2 (2.8%)	1 (4.0%)	>0.9
Thrombosis history	5 (6.9%)	1 (4.0%)	>0.9
Cardiovascular involvement	4 (5.6%)	3 (12%)	0.4

Continuous variables are expressed as median (interquartile range, IQR) and categorical variables as *n* (%).

**Table 3:** Univariable logistic regression analysis of factors associated with pregnancy loss history.

Variable	OR	95% CI	*p* Value
Gastrointestinal involvement	6.31	1.87–23.28	0.004
Ocular involvement	3.93	1.44–10.89	0.008
Cardiovascular involvement	2.32	0.43–11.31	0.295
CNS involvement	1.46	0.07–15.89	0.762
Skin involvement	1.35	0.54–3.41	0.515
Krause score	1.30	1.03–1.67	0.033
Disease duration	1.06	0.99–1.14	0.077
Age	1.04	0.96–1.13	0.334
Age at onset	0.97	0.90–1.03	0.347
Genital ulcer	0.72	0.29–1.84	0.485
Joint involvement	0.61	0.21–1.60	0.331
Thrombosis history	0.56	0.03–3.70	0.603
Oral ulcer	0.55	0.12–2.84	0.434

OR, odds ratio; CI, confidence interval.

## Data Availability

The data presented in this study are available on request from the corresponding author due to ethical restrictions.

## Supplementary Materials

The following supporting information can be downloaded at https://www.mdpi.com/article/10.3390/diagnostics16081133/s1. Table S1. Baseline characteristics of women with Behçet’s disease. Table S2. Clinical characteristics of women with Behçet’s disease according to pregnancy loss history. Table S3. Univariable logistic regression analysis of factors associated with pregnancy loss history.
